# Combining mHealth Technology and Pharmacotherapy to Improve Mental Health Outcomes and Reduce Human Rights Abuses in West Africa: Intervention Field Trial

**DOI:** 10.2196/53096

**Published:** 2024-03-27

**Authors:** Dror Ben-Zeev, Anna Larsen, Dzifa A Attah, Kwadwo Obeng, Alexa Beaulieu, Seth M Asafo, Jonathan Kuma Gavi, Arya Kadakia, Emmanuel Quame Sottie, Sammy Ohene, Lola Kola, Kevin Hallgren, Jaime Snyder, Pamela Y Collins, Angela Ofori-Atta, M-Healer Research Team

**Affiliations:** 1 Behavioral Research in Technology and Engineering Center, Department of Psychiatry and Behavioral Sciences, University of Washington, Seattle, WA, United States; 2 Department of Epidemiology, University of Washington, Seattle, WA, United States; 3 Department of Psychiatry, University of Ghana, Accra, Ghana; 4 Accra Psychiatric Hospital, Accra, Ghana; 5 Department of Psychiatry, University of Ibadan, Ibadan, Nigeria; 6 Institute of Psychiatry, Psychology & Neuroscience, King’s College London, London, United Kingdom; 7 Department of Human Centered Design and Engineering, University of Washington, Seattle, WA, United States; 8 Department of Mental Health, Bloomberg School of Public Health, John Hopkins University, Baltimore, MD, United States

**Keywords:** mHealth, mobile health, app, apps, applications, human rights, Africa, healers, healer, alternative, complementary, CAM, schizophrenia, bipolar disorder, depression, bipolar, depressive, mental, schizophrenic, schizophrenics, psychosocial, training, abuse, behavior change, behaviour change, medication, medications, pharmacology, pharmacological, pharmacotherapy, feasibility, acceptability, safety

## Abstract

**Background:**

In West Africa, healers greatly outnumber trained mental health professionals. People with serious mental illness (SMI) are often seen by healers in “prayer camps” where they may also experience human rights abuses. We developed “M&M,” an 8-week-long dual-pronged intervention involving (1) a smartphone-delivered toolkit designed to expose healers to brief psychosocial interventions and encourage them to preserve human rights (M-Healer app), and (2) a visiting nurse who provides medications to their patients (Mobile Nurse).

**Objective:**

We examined the feasibility, acceptability, safety, and preliminary effectiveness of the M&M intervention in real-world prayer camp settings.

**Methods:**

We conducted a single-arm field trial of M&M with people with SMI and healers at a prayer camp in Ghana. Healers were provided smartphones with M-Healer installed and were trained by practice facilitators to use the digital toolkit. In parallel, a study nurse visited their prayer camp to administer medications to their patients. Clinical assessors administered study measures to participants with SMI at pretreatment (baseline), midtreatment (4 weeks) and post treatment (8 weeks).

**Results:**

Seventeen participants were enrolled and most (n=15, 88.3%) were retained. Participants had an average age of 44.3 (SD 13.9) years and 59% (n=10) of them were male. Fourteen (82%) participants had a diagnosis of schizophrenia and 2 (18%) were diagnosed with bipolar disorder. Four healers were trained to use M-Healer. On average, they self-initiated app use 31.9 (SD 28.9) times per week. Healers watched an average of 19.1 (SD 21.2) videos, responded to 1.5 (SD 2.4) prompts, and used the app for 5.3 (SD 2.7) days weekly. Pre-post analyses revealed a significant and clinically meaningful reduction in psychiatric symptom severity (Brief Psychiatric Rating Scale score range 52.3 to 30.9; Brief Symptom Inventory score range 76.4 to 27.9), psychological distress (Talbieh Brief Distress Inventory score range 37.7 to 16.9), shame (Other as Shamer Scale score range 41.9 to 28.5), and stigma (Brief Internalized Stigma of Mental Illness Scale score range 11.8 to 10.3). We recorded a significant reduction in days chained (1.6 to 0.5) and a promising trend for reduction in the days of forced fasting (2.6 to 0.0, *P*=.06). We did not identify significant pre-post changes in patient-reported working alliance with healers (Working Alliance Inventory), depressive symptom severity (Patient Health Questionnaire-9), quality of life (Lehman Quality of Life Interview for the Mentally Ill), beliefs about medication (Beliefs about Medications Questionnaire–General Harm subscale), or other human rights abuses. No major side effects, health and safety violations, or serious adverse events occurred over the course of the trial.

**Conclusions:**

The M&M intervention proved to be feasible, acceptable, safe, and clinically promising. Preliminary findings suggest that the M-Healer toolkit may have shifted healers’ behaviors at the prayer camp so that they commit fewer human rights abuses.

## Introduction

Mental health systems in West Africa are constrained in their resources, infrastructure, and access to trained mental health personnel, limiting their capacity to deliver care [1,2]. In part due to these limitations, and in part due to local belief systems that frame mental health problems as spiritual rather than medical or psychological in nature [3,4], people with serious mental illness (SMI) in the region are often treated by traditional or faith healers, who are in abundance [5,6].

Healers often provide services at spiritual centers or “prayer camps”—rustic facilities where family members bring their relatives with mental illness, developmental disabilities, or substance use problems to be “healed” [7]. Pragmatically, prayer camps serve as West Africa’s de facto inpatient units and psychiatric residential homes. Once there, individuals seeking care may be retained at prayer camps for weeks, months, or even years at a time. Prayer camps are unregulated by any authorities, for the most part. Healers rarely have training in the etiology, assessment, or treatment of psychopathology, and their clients will seldom receive psychotropic medications or evidence-based psychosocial interventions. Healers provide spiritual consultation, prescribe prayer, and administer various ceremonial or herbal remedies [8,9]. In the absence of both training and resources to manage mental illness, healers may also engage in practices that have dangerous effects and constitute human rights abuses. These may include forced fasting, flogging, confinement in overcrowded or unsanitary conditions, and chaining patients to trees or concrete slabs so that they do not escape the camp’s grounds [9,10]. Chaining or similar forced mechanical restraining of people with SMIs, particularly those exhibiting signs of psychosis, are common practices in many low- and middle-income countries and are psychologically and physically damaging [11-13]. Despite the well-documented harsh practices used by some healers, and the negative attention their practices receive from Western media and human rights groups, these paraprofessionals continue to receive referrals and fill a societal need.

Our multinational team works closely with traditional and faith healers and people with SMI in West Africa. Through consultation, bilateral information sharing, articulation of mutual respect, and transparency in our activities, we have fostered partnerships with healers and their prayer camp communities [14-16]. These ties have served as the building blocks for cross-sector collaborations that are designed to improve the safety and quality of care that people with mental illness receive at prayer camps.

Findings from these collaborations have led us to develop M&M, a dual-pronged intervention involving (1) a smartphone-delivered mobile health treatment support toolkit (M-Healer app) designed to expose healers to brief psychosocial interventions, encourage them to preserve human rights in their practice, and prompt them to monitor the status of the people they serve, and (2) a visiting community nurse who provides pharmacological care directly to their patients at the prayer camp (Mobile Nurse). The combination of healer-facing support technology, which can be installed on mobile devices that are widely accessible to healers throughout the region, and patient-facing pharmacotherapy delivered by medically trained personnel, which are more widely available than specialty mental health providers, may be a scalable strategy for addressing unmet mental health and quality-of-care needs in the region. Here we report on the first field trial of the integrated M&M intervention in a prayer camp in Ghana.

## Methods

### Study Design

The study involved a field trial of the integrated M&M intervention at a large prayer camp in Ghana. The objectives of the study were to (1) examine the safety and acceptability of the intervention among people with mental illness who are treated at the camp (hereinafter referred to as “patients”) and the prayer camp staff who provide them with services (hereinafter referred to as “healers”) and (2) evaluate the preliminary effectiveness of the intervention package.

### Ethical Considerations

The study was approved by the institutional review boards of the University of Washington (00015549) and the University of Ghana (00001276). All study participants provided informed consent. Both healer and patient participants were compensated 75 Ghanaian Cedi (US $5.83) for their engagement in each assessment interview with study personnel.

### Study Procedures

Initially, members of the investigative team met with the leader of the prayer camp (hereinafter referred to as “the Prophet”) to describe the project (ie, objectives, procedures, and timeline), introduce members of the team, and demonstrate the M-Healer technology on a smartphone. During this meeting, the team was introduced to the 4 healers who would participate in the study. In a second meeting, the team met with the healers separately to discuss their routine practices at the camp, describe the “enhanced” services they would be trained to deliver in the context of the study, solicit feedback, cocreate the study’s operational timeline, and address any questions or concerns.

Candidate patients for intervention were suggested by prayer camp staff and screened on-site by study team personnel. The initial screening was conducted by study doctors (psychiatry residents). Participants were evaluated for general medical and psychiatric fitness for participation. Medical assessment included measurement of temperature, pulse, BMI, blood pressure, random blood sugar, blood hemoglobin level, and malaria. The study inclusion criteria were as follows: (1) being aged 18 years or older; (2) speaking English or Twi; (3) being a current inpatient staying at a study prayer camp; (4) having a diagnosis of schizophrenia spectrum disorder (ie, schizophrenia, schizoaffective disorder, delusional disorder, or schizophreniform disorder), bipolar disorder, or major depressive disorder, as determined by study staff administering the relevant sections of the Structured Clinical Interview for the *Diagnostic and Statistical Manual of Mental Disorders* (Fifth Edition) during screening. Candidates were excluded if they had a serious physical illness or needed urgent medical attention (eg, they had a high fever, serious infection, visible injury, or hemorrhage). Individuals who met the criteria and who were interested in participating were enrolled in the study. Participants were provided with a snack and meal by the study staff during assessment visits.

### Intervention Description

M&M is an 8-week-long dual-pronged intervention package comprising psychoeducation, skills training, and treatment support scaffolding tools delivered to healers via the M-Healer toolkit app, and pharmacotherapy that is administered directly to patients at prayer camps by a visiting mobile nurse.

#### Intervention Component 1: M-Healer Toolkit

**Figure 1.**
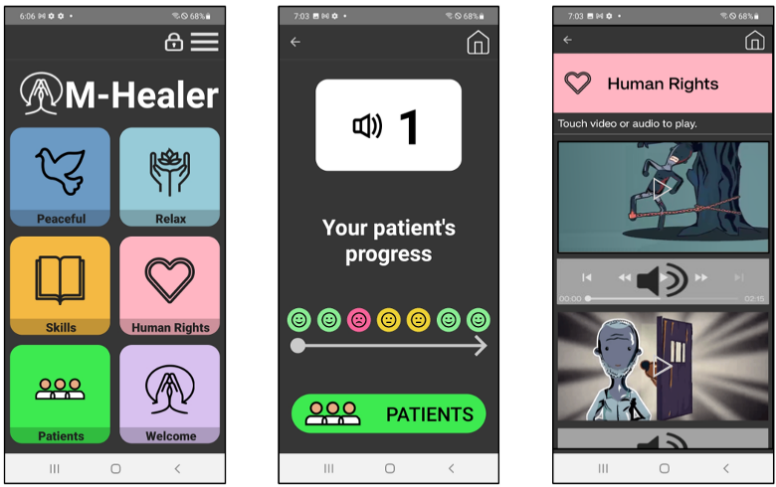
M-Healer home screen, patient check-in page, and on-demand human rights module.

M-Healer is an Android Smartphone app that was specifically designed to serve as a digital toolkit for healers providing care to people with mental illness in West Africa [16]. The goal of the toolkit is to provide exposure to psychoeducational materials and to support basic monitoring of patient progress. Once installed on an Android device, the app does not require an active data plan or internet connectivity to operate, mitigating possible disruptions in functionality in resource-constrained environments. M-healer is available in English and Twi, the 2 most common languages spoken in Ghana. The app has 4 core functionalities. First, M-Healer provides psychoeducation on the administration of psychosocial interventions such as guided relaxation techniques, rapport building, verbal de-escalation, challenging dysfunctional beliefs about psychiatric symptoms, and preservation of human rights and dignity in practice. All content is accessible “on demand” from the home screen and delivered as brief digital animations or audio recordings (Figure 1). Second, M-Healer allows healer users to create a list of active patients currently at the camp to support basic tracking and monitoring of individual progress. New patients are assigned a number, and healers have the option to also record an audio identifier for each individual. Every day, M-Healer prompts the healer to check in with each patient and provide a rating on whether they are doing better, worse, or the same as the day before. Ratings are assigned in the form of emojis with happy, sad, or neutral facial expressions. Once entered, a daily rating is added to a series of small icons showing patient progress over several days, which enables the healer to track patient progress over time (Figure 1). Third, M-Healer has push notifications, prompting users to watch the “video of the day” drawn from the bank of psychosocial digital animation training videos. Fourth, alongside these user-facing functionalities, M-Healer also tracks the frequency and duration of use in a given time frame, allowing the study staff and the nurse to monitor healers’ interactions with the M-Healer toolkit.

Healer participants were provided Android smartphones with M-Healer installed and activated, which they were allowed to retain at the conclusion of the study. Healers were trained by 2 practice facilitators (study coinvestigators) and the study nurse on how to use M-Healer. Practice facilitation was conducted over 2 sessions in a designated space within the prayer camp, which was removed from the areas where patients resided. In the first session, the practice facilitators established rapport and trust, introduced the M-Healer toolkit, and taught some technical and clinical skills to the healer. This session was interactive and involved demonstrations of how to use the system and its features. Each healer had an opportunity to practice these features with the assistance of the facilitators and the nurse. To help the healers understand how they could use M-Healer skills in caring for their patients, facilitators led role-plays, which involved the use of preselected videos and providing real-time instruction as the healers navigated through their devices. Role-playing skills in the videos involved the participation of both cofacilitators or a cofacilitator and a volunteer who modeled as a healer and patient. Role-playing sessions on how to add patients and track their progress involved all healers. As the lead facilitator led the training, the other study team members walked around to assist healers who were experiencing navigational or technical difficulties. To conclude the session, healers were tasked to watch a video, listen to an audio module, and check in and track the progress of their patients on a daily basis.

The second facilitation session was designed to strengthen rapport and trust, refine technical skills, and strengthen clinical skill acquisition. The facilitators and study nurse reviewed the healers’ experiences of using M-Healer and their use data. Healers’ efforts in using the app were also acknowledged and praised. Healers were then asked to demonstrate how to watch a video or listen to an audio module, add a new patient for daily monitoring, and record their patients’ progress. The facilitators addressed any questions and concerns. At the end of the session, the healers were reminded to watch a video, listen to an audio module, and check in with their patients daily moving forward. After these 2 training sessions, the healers continued to receive ongoing M-Healer support from the study nurse during weekly site visits. During these visits, the nurse had opportunities to interact with the healers, inquire about and address their difficulties, check and record their user data, and encourage their use of the app.

#### Intervention Component 2: Mobile Nurse

A registered mental health nurse trained to use the World Health Organization’s Mental Health Gap Action Programme–Intervention Guide (mhGAP-IG; version 2.0) served as the intervention’s mobile nurse. Participants were prescribed medications by the screening study doctors. When possible, the prescribers opted to recommend long-acting injectable medications for participants with symptoms of psychosis to mitigate the risk of poor adherence to oral medication regimens. The use of long-acting injectable medications was facilitated by the fact that most of the participants had received treatment with antipsychotics prior to their entry into the camp. Participants ultimately decided whether they would take medication and specified their preferred medication modality. The nurse monitored participants’ BMI, blood pressure, pulse, and temperature weekly and monitored their random blood sugar monthly. The nurse conducted weekly assessments of response to treatment and medication side effects using mhGAP-IG guidance. The nurse was supervised clinically by 2 study psychiatrists and instructed to call them if they encountered any urgent treatment-related problems that were not clearly described by the mhGAP-IG or the study protocol. The clinical team maintained active, real-time telecommunication to facilitate timely consultation and a weekly clinical team meeting to review nonurgent inquiries.

### Data Collection

Study assessors (masters’-level clinical psychologists) administered a battery of measures to patient participants at baseline, midintervention (week 4), and post intervention (week 8). Assessments were administered in English or Twi using a tablet-based data collection software REDCap (Research Electronic Data Capture) to facilitate secure, password-protected, on-site digital data collection. We collected information on demographics, psychological symptoms, psychological distress, quality of life, and prayer camp experiences.

Patient participants’ psychiatric symptoms were measured at baseline, midtreatment, and post treatment using three measures: (1) the Brief Psychiatric Rating Scale (BPRS) [17], an 18-item clinical assessor–rated measure of current indicators of psychiatric illness including somatic concern, anxiety, emotional withdrawal, conceptual disorganization, feelings of guilt, tension, mannerism and posturing, grandiosity, depressive mood, hostility, suspiciousness, hallucinatory behavior, motor retardation, uncooperativeness, unusual thought content, blunted affect, excitement, and disorientation; the study assessors rated each indicator from 1 (“not present”) to 7 (“extremely severe”), with higher scores indicating more severe symptoms (range 18-126); (2) the Brief Symptom Inventory (BSI) [18], a 53-item self-report assessment measuring 9 symptom dimensions; patients rated how much each symptom has bothered them in the past 8 days from 0 (“not at all”) to 4 (“extremely”), with higher scores indicating more severe distress over the past 7 days (range 0-212); and (3) the Patient Health Questionnaire-9 [19], a 9-item depressive symptom scale in which respondents self-report how often (1=“not at all” to 4=“nearly every day”) they experience symptoms including feeling depressed and hopeless over the prior 2 weeks.

The following measures were also administered at baseline, midtreatment, and post treatment. Psychological distress was measured using the Talbieh Brief Distress Inventory [20], a 24-item self-report measure using a Likert scale for prior-month frequency rating (0=“not at all” to 4=“extremely”). Higher scores represent more severe distress. Quality of life was measured using the global quality-of-life items from the Lehman Quality of Life Interview for the Mentally Ill [21], whereby participants rate how they feel about various aspects of their life from 1 (“terrible”) to 7 (“delighted”), and higher scores indicate higher quality of life. Shame was measured using the Other as Shamer Scale [22], an 18-item self-report (range 8-32; higher scores correspond to higher experiences of guilt and shame). Patient beliefs about medication were assessed using the Beliefs about Medications Questionnaire–General Harm subscale [23], internalized stigma was measured with the Brief Internalized Stigma of Mental Illness scale [24], and patient working alliance with the healers at the camp was assessed with an adapted version of the Working Alliance Inventory [25].

Human rights abuses were measured at baseline, midtreatment, and post treatment. At each assessment, study assessors interviewed patient participants and asked them to self-report on their experiences during the preceding week, up until and including the day of the assessment. Specially, they were asked to report the number of days they were chained or shackled (ie, “days chained”), the number of days they were forced to take herbal remedies, the number of hours they were held in isolation, and the total number of times they were touched on their genitals in an uncomfortable manner in the previous week.

To characterize the sample before treatment, we collected data on individuals’ history of sexual abuse (Sexual Abuse Severity Scale) [26] substance use (Tobacco, Alcohol, Prescription Medications, and Other Substances scale) [27], and social support (Oslo Social Support Scale) [28] at baseline.

To measure the feasibility of the M-Healer intervention, we collected data post treatment on healers’ use of the M-Healer technology, as well as knowledge and skills related to engagement with M-Healer. At each visit, study staff viewed the password-protected M-Healer data analytics screen on the healers’ phones and recorded data including the number of days on which the app was used, the number of videos watched, the number of user-initiated app interactions, and the number of prompts to which the healers responded. To assess fidelity, healers participated in a 12-item verbally administered knowledge checklist whereby healers agreed or disagreed with statements regarding M-Healer knowledge domains. Healers performed 3 behavioral tests to demonstrate skills learned in M-Healer modules by interacting with the study staff acting as a standardized patients in role-played scenarios common to prayer camp settings. Study staff rated healer-demonstrated skills in a checklist.

### Statistical Analysis

Descriptive statistics were used to characterize patient participants and their psychiatric and psychosocial factors at all study visits. We used paired samples *t* tests to compare baseline to midtreatment and baseline to posttreatment mean scores. For participants missing <50% of scale items for a psychosocial scale, item-level scores were imputed as the median score across the participant’s existing scale items (person-median imputation) [29]. Psychosocial scores were not analyzed for participants missing ≥50% of scale items. Quantitative analyses were conducted using Stata (version 17; StataCorp).

## Results

**Figure 2.**
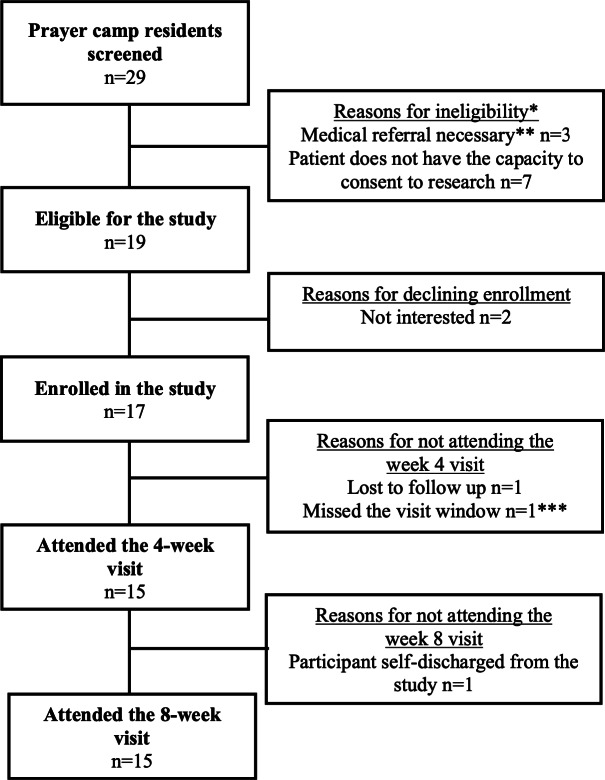
Flowchart for prayer camp participant enrollment. *Reasons for ineligibility were not mutually exclusive. **Malaria Rapid Diagnostic Test positive: n=1; hemoglobin level < 8% and symptoms: n=1; pulse > 120 bpm and symptoms: n=2. ***Participants returned for the 8-week visit.

In total, 29 patients were screened for study eligibility among whom 19 (65.5%) were eligible to participate and 17 (89.5%) were enrolled (Figure 2). The majority of participants (n=15, 88.3%) were retained for midtreatment (4 weeks) and posttreatment (8 weeks) follow-up visits. Participants had an average age of 44.3 (SD 13.9) years; 59% (n=10) of them were male, and 82% (n=14) of them identified as Christian (Table 1). Fourteen (82%) participants had a diagnosis of schizophrenia, 1 had a comorbid diagnosis of major depressive disorder, and 2 were diagnosed with bipolar disorder. The average number of weeks spent residing in the camp was 72.6 (SD 111.9), and this was the first experience in a prayer camp for 41% (n=7) of participants.

We found a significant and clinically meaningful reduction in psychiatric symptom severity from pretreatment to posttreatment as measured by the BPRS and the BSI (Table 2). Subjective ratings of psychological distress and shame were significantly lower post treatment than before treatment. Participants had significantly reduced internalized stigma about their mental health conditions from pretreatment to posttreatment. We recorded a significant reduction in days chained from pretreatment to posttreatment.

**Table 1.** Study sample characteristics (N=17).

Demographic characteristics		Value
Age (years; n=16), mean (SD)		44.3 (13.9)
**Sex, n (%)**		
	Female	7 (41.0)
	Male	10 (59.0)
**Educational attainment, n (%)**		
	Primary	2 (11.8)
	Junior high school	6 (35.3)
	Senior high school	5 (29.4)
	Tertiary or higher	4 (23.5)
**Marital status, n (%)**		
	Married	6 (35.0)
	Divorced or separated	2 (12.0)
	Single	9 (53.0)
**Religion n (%)**		
	Christian	14 (82.0)
	No religion	1 (6.0)
	Other	1 (6.0)
	Missing	1 (6.0)
Weeks residing in this prayer camp (n=14), mean (SD)		72.6 (111.9)
**First experience residing in a prayer camp**		
	Yes	7 (41)
	No	8 (47)
	Missing data	2 (12)
Social support^a^ (n=12), mean (SD)		7.8 (2.6)
Ever use tobacco^b^, n (%)		2 (12)
Ever use alcohol^b^, n (%)		2 (12)
Ever use drugs^b^, n (%)		2 (12)
Ever use prescription drugs^b^, n (%)		0 (0)
**Psychiatric diagnosis n (%)**		
	Schizophrenia	14 (82.0)
	Bipolar disorder	2 (12.0)
	Major depressive disorder (comorbid with a primary diagnosis of schizophrenia)	1 (6.0)

a 3-item Oslo Social Support Scale; absolute range 3-12; a higher score indicates a higher level of social support.

b Tobacco, alcohol, prescription medications, and other substances scale.

**Table 2.** Summary of M&M treatment outcomes.

	Pretreatmentbaseline assessment (n=17)		Midtreatment 4-weekassessment (n=15)			Posttreatment 8-week assessment (n=15)		
	N	Mean (SD)	N	Mean (SD)	*P* value	N	Mean (SD)	*P* value
BPRS^a^	16	52.3 (24.0)	15	41.4 (10.6)	*.01^b^*	15	30.9 (8.7)	*<.001*
BSI^c^	16	76.4 (56.6)	14	53.9 (42.7)	*.004*	13	27.9 (23.7)	*<.001*
TBDI^d^	15	37.7 (23.5)	13	27.8 (20.9)	*.002*	14	16.9 (12.9)	*<.001*
OAS^e^	15	41.9 (21.3)	12	35.1 (20.6)	.64	13	28.5 (21.5)	*.01*
BMQ-Harm^f^	14	11.8 (3.6)	12	10.0 (4.1)	*.007*	12	10.3 (4.9)	.40
ISMI-10^g^	14	30.4 (3.9)	—^h^	—	—	13	24.9 (5.9)	*<.001*
PHQ-9^i^	15	10.4 (6.5)	14	9.2 (7.9)	.35	15	8.5 (8.5)	.21
Lehman Quality of Life Scale for the Mentally Ill^j^	14	20.1 (7.8)	11	23.5 (10.0)	.40	15	22.5 (9.2)	.26
Working Alliance Inventory–Bond Subscale^k^	13	14.8 (5.6)	11	13.8 (5.1)	.80	12	15.4 (3.9)	.59
Days chained^l^	16	1.6 (2.8)	14	0.5 (1.9)	.09	15	0.5 (1.8)	*.047*
Days forced to fast^l^	16	2.6 (5.5)	14	0.3 (1.1)	.12	15	0.0 (0.0)	.06
Days forced to take herbal remedies^l^	16	0.06 (0.3)	12	0.6 (2.0)	.40	15	0.0 (0.0)	.32
Hours kept isolated^l^	16	3.0 (8.2)	14	0.0 (0.0)	.32	15	0.0 (0.0)	.14
Times touched in an uncomfortable manner^l^	16	0.1 (0.3)	14	0.0 (0.0)	.14	15	0.0 (0.0)	.14

a BPRS: Brief Psychiatric Rating Scale—an 18-item scale with an absolute range of 18-126; higher scores indicate higher symptom severity.

b Italicized values are statistically significant at *P*<.05.

c BSI: Brief symptom inventory—a 53-item scale with an absolute range of 0-212; higher scores indicate higher symptom severity.

d TBDI: Talbieh Brief Distress Inventory—a 24-item scale with an absolute range of 0-96; higher scores indicate greater distress.

e OAS: Other as Shamer Shame Scale—an 18-item scale with an absolute range of 0-90; higher scores indicate greater shame.

f BMQ-Harm: Beliefs about Medications Questionnaire–Harm Subscale—a 4-item scale with an absolute range of 4-20; higher scores indicate unfavorable thoughts about medications.

g ISMI-10: Internalized Stigma of Mental Illness Inventory—a 10-item scale with an absolute range of 10-40; higher scores indicate greater internalized stigma.

h Not available.

i PHQ-9: Patient Health Questionnaire-9—a 9-item scale with an absolute range of 0-27; higher scores indicate higher symptom severity.

j A 6-item scale with an absolute range of 6-42; higher scores indicate better quality of life.

k A 4-item scale with an absolute range of 4-20; higher scores indicate better working alliance.

l Frequency over the past week.

Psychiatric symptoms measured with the BPRS and BSI were reduced midtreatment compared to those at baseline. Unfavorable beliefs about medications had decreased by the midtreatment visit compared to those at baseline. We observed a trend toward statistical significance for reduction in forced fasting by the posttreatment follow-up visit (*P=*.06). Over the intervention period, we did not identify changes in patient-reported working alliance with healers, depressive symptom severity, quality of life, days forced to take herbal remedies, hours retained in isolation, or number of times a participant reported being touched in an uncomfortable manner.

Results with imputed variables were not meaningfully different from those obtained from raw data. Notably, a significant decrease in unfavorable beliefs about medications from pretreatment to posttreatment was identified in the nonimputed data set; yet, this change was not detected in the imputed data set. Participants’ scales with imputed values were predominantly only missing 1-2 items, which were imputed using person-median values per scale to retain the available data.

In the initial visit to the prayer camp, 15 participants consented to receiving pharmacotherapy. For schizophrenia treatment, fluphenazine decanoate (25 mg/mL) was the most commonly prescribed pharmacotherapy (n=12, 80.0%), followed by trihexyphenidyl (5 mg tablet; n=6, 40.0%) and risperidone (2 mg; n=3, 20.0%); some participants were prescribed multiple medications. Among participants with bipolar disorder, fluphenazine decanoate (25 mg), risperidone (25 mg), and trihexyphenidyl (5 mg) were each prescribed to 6.7% (n=1) of participants. Overall, 110 medication follow-up visits were conducted by the mobile nurse.

Four healers were trained to use M-Healer and their usage of the app was monitored weekly. M-Healer feasibility measures indicated that healers had an average of 31.9 (SD 28.9) user-initiated app uses per weekly visit, watched 19.1 (SD 21.2) videos, responded to 1.5 (SD 2.4) prompts, and used the app for 5.3 (SD 2.7) days a week. Healers had posttreatment average knowledge assessment checklist scores of 11.75 (absolute range 9-14, total possible score=16). Two healers successfully demonstrated all 3 behavioral skills, 1 healer demonstrated 2 skills, and 1 healer demonstrated 1 skill.

## Discussion

### Principal Results

The combination treatment package deployed in this study was designed to inform and shape West African healers’ practices and to provide symptomatic relief to individuals with psychiatric illnesses receiving services at their prayer camps. The intervention proved to be feasible and acceptable to healers and their patients. Healers were able to navigate the M-Healer system successfully on the study smartphone devices that were provided to them. Healers expressed a clear understanding of M-Healer functionalities, watched psychoeducational videos, and listened to the audio lessons over the study period. Healers in the study self-initiated M-Healer use throughout the intervention period above and beyond system-prompted interactions and chose to view M-Healer videos and listen to audio lessons at a rate that far exceeded our expectations. Healers’ use of the app was very frequent—almost daily. When evaluated, healers were able to describe the content accurately and to demonstrate skills that they had learned from the app.

Healers agreed to grant the mobile nurse access to the individuals they treated at the prayer camp, the majority of whom were placed in locked dormitories, with some shackles. The nurse was able to assess, provide pharmacotherapy to, and monitor these patients weekly without difficulty or obstruction, despite these austere conditions. All patients approached by our study team expressed openness to meeting with the nurse, and all but 1 expressed interest in receiving pharmacotherapy to manage their psychiatric symptoms and improve their health.

The combined intervention proved to be clinically promising. Patient participants experienced significant reductions in the severity of their psychiatric symptoms, psychological distress, subjective feelings of shame, and internalized stigma over the course of the intervention. Moreover, patient participants reported a significant reduction in the days during which they were chained or shackled by the healers and a nonsignificant trend of reduction in the days during which they were forced to fast. These findings suggest that exposure to the training content delivered by the M-Healer toolkit may have shifted healer behaviors after the intervention was implemented at the prayer camp’s study site. One participant was discharged from the prayer camp and allowed to return to their family over the course of the intervention by prayer camp staff.

The intervention proved to be safe. The study helped promote better care in the prayer camp’s study site. Over the course of the intervention, 3 participants were identified by our study staff as requiring immediate medical attention and were referred to the district hospital where they received care. The nurse monitored medication side effects weekly. No major side effects, health and safety violations, serious adverse events, or other major complications occurred over the course of the field trial.

Poststudy debriefing interviews with healers indicated that they felt some ambivalence about M-Healer content. On one hand, they enjoyed the opportunity to learn about novel psychosocial illness management strategies and indicated that the digital toolkit informed the way they engaged with their patients. On the other, some expressed frustration that the toolkit’s human rights module seemed too critical of their practices (eg, M-Healer’s guidance to refrain from chaining patients). Given that current prayer camp practices do not prioritize preservation of human rights, this tension may have been inevitable. A more complete report on the findings from these interviews will be provided in a separate qualitative study.

### Limitations

The study has several limitations. First, the study involved patient participants and healers at a single prayer camp, limiting study generalizability. Prayer camps vary dramatically in terms of their size, practices, and openness to research and outsider involvement in their day-to-day activities. Future multisite research in several prayer camps can help determine the generalizability of our findings to other settings. Second, we relied on study participants’ self-reports to determine whether they experienced human rights abuses at the camp. Participants may have underreported these events due to shame, fear of retaliation, or to protect camp staff. Third, the study sample was small, allowing only for preliminary examination of treatment effects. Despite the exploratory nature of our evaluation of outcomes, promising evidence of clinical effectiveness was recorded. Future research involving larger samples will facilitate more robust evaluation of the effects of the intervention on participants’ health and well-being. Finally, prayer camp staff had previous experience being involved in research with our team. It will be important to examine the intervention in research-naïve prayer camps to evaluate whether such relationships are necessary for successful intervention uptake and outcomes.

### Conclusions

In West Africa, traditional and faith healers greatly outnumber trained mental health professionals. Healers hold political capital and are respected by the local population. These key stakeholders can serve as facilitators, conduits, obstructionists, or blockades to the delivery of evidence-based mental health services in their communities. Engaging healers and training them to provide compassionate psychosocial interventions themselves and opening the doors of their prayer camps to medical professionals who can also deliver pharmacological interventions have the potential to enhance the regional capacity to address unmet mental health needs.

Previous research has demonstrated that pharmacotherapy can be conducted successfully at prayer camps. While psychotropics may help effectively manage the psychiatric symptoms of patients staying at prayer camps, those improvements did not translate to reductions in the days during which patients were shackled [14]. This study demonstrated that adding a key component—a healer-facing digital health training toolkit—can impact how healers interact with their patients, including a reduction in the use of mechanical restraints. These findings are encouraging and suggest that the M&M intervention holds promise as a dual-pronged model for both improving mental health outcomes and reducing human rights abuses in West African prayer camps. If future research finds similarly robust treatment effects on a larger scale, this will provide important information for Ghanian policy makers. Such findings would demonstrate that a combination of accessible technologies coupled with individuals trained to deliver interventions in the field may help reconfigure how prayer camps serve people with SMI. Ghanaian government mandates, monitoring, and enforcement over the last few years are beginning to produce positive effects on the reduction of human rights abuses in prayer camps. In addition to barring harmful practices, the government can also play a key role in supporting the adoption of new models of care. Through targeted trainings and appropriate oversight, individuals who are already working in the field (eg, government-employed district nurses or members of the Psych Corps—psychology graduates who are posted to health facilities across the country as part of their national service) could be leveraged to train healers and other paraprofessionals in the use of evidence-based digital mental health technologies. Government investment in such force-multiplying activities and technologies can prove to be a pragmatic and scalable approach for addressing significant professional workforce shortages in the region.

## Abbreviations

BPRS: Brief Psychiatric Rating Scale
BSI: Brief Symptom Inventory
mhGAP-IG: Mental Health Gap Action Programme–Intervention Guide
REDCap: Research Electronic Data Capture
SMI: serious mental illness
